# Merlin immunoreactivity fails to predict *neurofibromatosis type 2* mutations in human meningiomas

**DOI:** 10.1093/jnen/nlaf058

**Published:** 2025-05-30

**Authors:** Sofie Eline Tollefsen, Rahmina Meta, Ole Solheim, Patricia Mjønes, Ingfrid Vestrheim, Wenche Sjursen, Sverre Helge Torp

**Affiliations:** Department of Clinical and Molecular Medicine, Faculty of Medicine and Health Sciences, Norwegian University of Science and Technology (NTNU), Trondheim, Norway; Department of Clinical and Molecular Medicine, Faculty of Medicine and Health Sciences, Norwegian University of Science and Technology (NTNU), Trondheim, Norway; Department of Neurosurgery, St Olavs Hospital, Trondheim University Hospital, Trondheim, Norway; Department of Neuromedicine and Movement Science, Faculty of Medicine and Health Sciences, Norwegian University of Science and Technology (NTNU), Trondheim, Norway; Department of Clinical and Molecular Medicine, Faculty of Medicine and Health Sciences, Norwegian University of Science and Technology (NTNU), Trondheim, Norway; Department of Pathology, St Olavs Hospital, Trondheim, Norway; Department of Clinical and Molecular Medicine, Faculty of Medicine and Health Sciences, Norwegian University of Science and Technology (NTNU), Trondheim, Norway; Department of Clinical and Molecular Medicine, Faculty of Medicine and Health Sciences, Norwegian University of Science and Technology (NTNU), Trondheim, Norway; Department of Medical Genetics, St Olavs Hospital, Trondheim, Norway; Department of Clinical and Molecular Medicine, Faculty of Medicine and Health Sciences, Norwegian University of Science and Technology (NTNU), Trondheim, Norway; Department of Pathology, St Olavs Hospital, Trondheim, Norway

**Keywords:** brain tumor, diagnosis, immunohistochemistry, meningioma, merlin, *NF2* mutation

## Abstract

Deletion in 22q and mutations in the *neurofibromatosis type 2 (NF2)* gene are frequent in sporadic meningiomas. The tumor suppressor protein merlin is encoded by *NF2*, and mutations may promote tumor development. *NF2* status is increasingly important in meningioma diagnostics and we questioned whether merlin immunohistochemistry could be used as an accessible and affordable surrogate marker for prediction of *NF2* mutations. Previous studies on merlin immunoreactivity have reported diverging results. We aimed to describe the immunohistochemical expression of merlin in a large series of meningiomas and relate these findings to clinicopathological features and *NF2* status. Standardized immunohistochemistry was conducted on 172 meningiomas using three different merlin antibodies directed toward the N-terminal, C-terminal and phospho-merlin (ser 518). Twenty of the included cases had known *NF2* status. All tumor specimens were immunoreactive for the three merlin antibodies. The immunoreactivity of phosphorylated merlin was higher in meningothelial tumors. There were no other significant associations between merlin immunoreactivity and *NF2* status, WHO grade, tumor subtype, tumor location or gender. These results indicate that merlin immunoreactivity does not seem to be predictive of *NF2* mutation, as merlin was abundantly expressed by all included tumors and independently of *NF2* status.

## INTRODUCTION

Meningiomas are classified according to the World Health Organization (WHO) classification of tumors of the central nervous system (CNS).^1^ The classification system is based on an overall subjective assessment of histopathological criteria. In the updated WHO CNS classification from 2021, a limited number of molecular markers with importance in respect of grading and prognosis have been included. These markers include *TERT* promoter mutation and homozygous deletion of *CDKN2A/B*, which both are diagnostic criteria for WHO CNS grade 3 meningiomas. *Neurofibromatosis type 2 (NF2)* gene mutation and/or 22q deletion are frequent in meningiomas, but are not yet included in the WHO classification.^1^^,^^2^ The latest update from consortium to inform molecular and practical approaches to CNS tumor taxonomy-not official WHO (cIMPACT-NOW) suggest that meningiomas with 1p deletion and concurrent *NF2* mutations and/or 22q monosomy should be graded at minimum as CNS WHO grade 2 tumors.^3^

The *NF2* gene, located on chromosome 22q12, encodes the tumor suppressor protein merlin/schwannomin.^4^ Merlin consists of three domains (N-terminal, C-terminal, and central helix), and may appear in two activation states where the phosphorylation of the C-terminal residues provides an active state of the protein.^5^^,^^6^ This scaffold protein is crucial for multiple cell functions, such as linking receptors to the cytoskeleton and modulating several signaling pathways that control proliferation.^4^ Alterations in *NF2* are reported in more than half of meningiomas, suggesting an essential role of this gene for meningioma development.^2^^,^^4^^,^^7^ Studies have shown that 22q deletions and *NF2* mutations are more common in transitional and fibrous meningiomas, and with higher malignancy grade.^1–4^ Furthermore, low merlin expression has been suggested as predictive of sensitivity for FAK inhibitors. The recent alliance A071401 study report an improved progression-free survival at 6 months after FAK inhibition compared to historical controls.^8^

Previous studies have found variable merlin expression in human meningiomas.^9–15^ Accordingly, there is conflicting evidence as to whether merlin could be used as a surrogate marker for *NF2* gene mutations in meningiomas. Due to the prognostic importance of these mutations and need for objective biomarkers in meningioma diagnostics, it is relevant to identify practical and affordable methods to detect molecular genetic aberrations, such as *NF2* gene mutations. Next-generation sequence and methylation analyses are suitable, yet costly and effort-intensive analyses to apply. Immunohistochemical analyses are reasonable and easily accessible at most laboratories.^16^

We hypothesized that merlin immunoreactivity could be used as a surrogate marker of *NF2* mutations, providing a more accessible and affordable method for detecting these essential mutations. Accordingly, the aim of this study was to assess merlin immunoreactivity and relate these findings to clinicopathological features in a large series of human meningiomas. Three different antibodies for merlin targeting the N-terminal (AB-N), C-terminal (AB-C), and the phosphorylated protein (AB-P), were applied. Tumors with known *NF2* gene status were also included and their immunoreactivity for merlin was correlated to *NF2* gene status and 1p deletion.

## METHODS

### Patients

A total of 172 adult patients operated for primary intracranial WHO 2016 grade 1 or 2 meningioma were included. Among these, 162 patients were operated in the period of January 1, 1991, to December 31, 2000, at St Olavs Hospital, Trondheim University Hospital, Trondheim, Norway. These tissue specimens were prepared as tissue microarrays (TMAs), as previously described by Arnli et al.^17^ Next-generation sequencing was performed on 20 intracranial WHO grade 2 meningiomas, comprising 10 cases from the TMA specimens and 10 additional cases, as previously reported by Meta et al.^18^ The additional 10 patients were operated in the period of January 1st, 1991, to December 31st, 2004.

### Immunohistochemistry

Paraffin sections of 4 µm were dried overnight, then heated to 60°C before deparaffinization and rehydration. Heat-induced epitope retrieval was conducted by PT Link (DAKO Denmark A/S). Immunodetection was performed using Dako Autostainer Plus. Primary antibodies are listed in Table 1. After incubation of primary antibodies, Dako REAL Peroxidase Blocking Solution was applied to prevent endogenous peroxidase activity. Secondary antibodies were incubated. DAB+ chromogen (Dako REAL Envision Detection System) was applied and sections underwent hematoxylin counterstain. Tissue specimens from liver and cerebellum were used as positive controls. The primary antibodies were omitted for negative controls. All TMA cores were scanned utilizing Olympus VS120S5 with a ×20 objective lens.

**Table 1.** Primary antibodies.

Antibody	Producer	Type	Epitope	Dilution	Incubation time
Anti-NF2/merlin (LS-C164143)	LSBio	Polyclonal	N-terminal	1:100	1 h at room temperature
Anti-NF2/merlin (ab84550)	Abcam	Polyclonal	C-terminal	1:100	Overnight at 4 °C
Anti-NF2/merlin (ab2478)	Abcam	Polyclonal	Phosphorylated merlin (ser 518)	1:400	Overnight at 4 °C

### Scoring of immunohistochemistry

The immunoreactivity of each tumor was scored as: 0—negative, 1—weak, 2—moderate, or 3—strong. TMA cores with less than 50% remaining material were excluded, and each tumor needed more than one preserved TMA core to be evaluated. One collective immunoreactivity score was obtained for each patient. The scoring of immunohistochemistry was conducted by a trained medical doctor (R.M.) and supervised by an experienced neuropathologist (S.H.T.).

### Statistical analysis

Mann-Whitney *U* test was used to investigate the association of merlin immunoreactivity to *NF2* status and 1p deletion for the 20 included cases with known *NF2* status. Only TMA specimens were included in the further statistical analyses. Spearman’s rank-order correlation was used to determine correlation between the immunoreactivity of AB-N and AB-C. Mann-Whitney *U* was applied to investigate the relation of merlin immunoreactivity to the dichotomous categorical variables WHO 2016 classification (grade 1 vs grade 2) and gender (female vs male). Kruskal-Wallis test was used to investigate associations to tumor location and tumor subtype, and if significant followed by Dunn’s test with Bonferroni correction. Only tumor locations and tumor subtypes with more than one patient were included. The included tumor locations were as follows: (1) falcine, (2) convexity, (3) skull base, and (4) fossa posterior and tentorial. Included tumor subtypes were as follows: (1) meningothelial, (2) fibrous, (3) transitional, and (4) atypical. *P*<.05 was considered significant. IBM SPSS statistics version 29 was utilized for all statistical analyses.

### Ethical approval

This study was conducted according to the principles of the Declaration of Helsinki and a waiver of consent was given by the Regional Committees for Medical and Health Research Ethics (project number 4.2006.947).

## RESULTS

### Patient characteristics

Among the 162 included patients with tumor specimens prepared as TMA cores, 109 patients (67.3%) suffered from a WHO 2016 grade 1 meningioma. Median age at time of operation for these patients was 60 years, ranging from 25 years to 86 years. Most patients were female (73.3%). Transitional meningiomas (40.7%) were most frequent, followed by atypical (32.7%), meningothelial (17.3%), and fibrous (5.6%) tumors. Most tumors (49.4%) were at the convexity of the brain, while the skull base (22.2%) was the second most frequent tumor location. Patient data is shown in Table 2. The study also included 20 patients with known *NF2* status, comprising 10 patients from the TMA specimens and 10 additional cases. These 20 patients had a median age of 65, ranging from 36 to 86, and a female predomination (70% females). Most of the tumors were convexity meningiomas (80%). *NF2* mutations were found in 55% of the tumors. The identified mutations were frameshift, nonsense, and splice site mutation, all interpreted to cause loss of NF2 protein function. 1p deletion was detected in 40% of the tumors. Patient data with associated molecular-genetic alterations are described in Table 3.

**Table 2.** Patient characteristics for the 162 included patients with tumor specimens prepared as TMA cores.

All patients, *n*	162
Age at operation, median [range]	60 [25-86]
WHO grade, *n* (%)	
WHO grade 1	109 (67.3%)
WHO grade 2	53 (32.7%)
Gender, *n* (%)	
Female	119 (73.5%)
Male	43 (26.5%)
Tumor subtype^a^, *n* (%)	
Meningothelial	28 (17.3%)
Fibrous	9 (5.6%)
Transitional	66 (40.7%)
Atypical	53 (32.7%)
Tumor location, *n* (%)	
Skull base	36 (22.2%)
Convexity	80 (49.4%)
Falcine	24 (14.8%)
Fossa posterior and tentorial	21 (13.0%)
Intraventricular	1 (0.6%)

a Only included tumor subtypes with greater than 1 patient.

**Table 3.** Patient data with associated molecular-genetic alterations for the 20 tumors with known *NF2* status.

Gender	Age	Tumor location	CNV (1p deletion, 22q loss)	*NF2* mutation	DNA (protein), mutation type	AB-N	AB-C	AB-P
Male	47	FPT	1p, 22q	Yes	c.837_838delAA, p.(Lys279AsnfsTer14), frameshift mutation	3	3	3
Male	47	Convexity	1p, 22q	Yes	c.1396C > T, p.(Arg466Ter), nonsense mutation	2	2	2
Female	54	Convexity	1p, 22q	Yes	c.1093G>T, p.(Glu365Ter), nonsense mutation	3	3	3
Female	65	Convexity	1p, 22q	Yes	c.543_544delGGinsTT, p.(Glu182Ter), frameshift mutation	2	3	2
Female	66	Convexity	1p, 22q	Yes	c.1575-6C>A, p.(?)^a^	2	3	2
Female	36	Convexity	22q	Yes	c.297delA, p.(Lys99AsnfsTer24), frameshift mutation	2	3	2
Female	49	Convexity	22q	Yes	c.114 + 3A>C, p.(?)^b^	2	2	2
Female	72	Convexity	22q	Yes	c.432C>G, p.(Tyr144Ter), nonsense mutation	3	3	3
Female	75	Convexity	22q	Yes	c.1048delG, p.(Glu350AsnfsTer14), frameshift mutation	n/a^c^	2	n/a^c^
Male	83	Convexity	22q	Yes	c.448-1G > C, p.(?), splice site mutation	2	3	2
Male	86	Convexity	22q	Yes	c.467_473delGTGTTCA, p.(Ser156ThrfsTer16), frameshift mutation	2	2	3
Female	71	Convexity	1p, 22q	No	*NF2* wildtype	2	3	2
Female	72	Skull base	1p, 22q	No	*NF2* wildtype	n/a^c^	3	n/a^c^
Male	38	Convexity	1p	No	*NF2* wildtype	3	2	3
Female	42	Convexity	22q	No	*NF2* wildtype	2	2	3
Female	46	Convexity	22q	No	*NF2* wildtype	2	1	1
Male	65	Falcine	22q	No	*NF2* wildtype	2	3	1
Female	80	Skull base	22q	No	*NF2* wildtype	3	2	3
Female	60	Convexity	None detected	No	*NF2* wildtype	3	3	3
Female	67	Convexity	None detected	No	*NF2* wildtype	2	3	3

Copy number variant (CNV) with 1p deletion and/or 22q loss are described. DNA (protein) and mutation type are reported for the *NF2*-mutated tumors, with the remaining being *NF2* wildtype tumors. Scorings of merlin immunoreactivity for all three applied antibodies (AB-N, AB-C, and AB-P) are provided. *NF2* mutation transcript: NM_000268.3.

a Frameshift mutation due to new stronger splice site, resulting in the inclusion of 4 intronic nucletotides (−98% by MaxEntScan, NNSPLICE and SpliceSiteFinder-like).

b Mutation presumed to disrupt donor splice site (−93% by MaxEntScan, NNSPLICE and SpliceSiteFinder-like).

c Not assessed due to lack of tissue (n/a).

Abbreviations: AB-C, anti-NF2/merlin ab84550; AB-N, anti-NF2/merlin LS-C164143; AB-P, anti-NF2/merlin ab2478; CNV, copy number variant; FPT, fossa posterior and tentorial.

### Immunohistochemical expression of merlin

**Figure 1.**
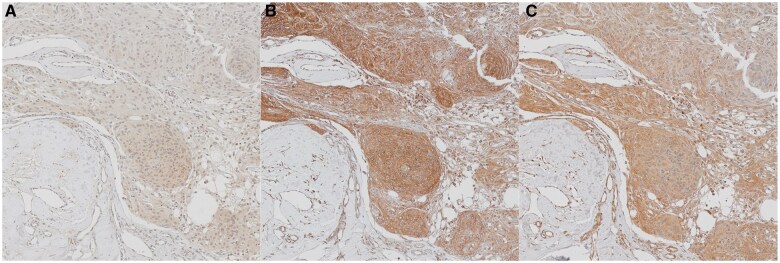
Immunohistochemical imaging of merlin immunoreactivity from the same tumor specimen, with fibrosis as internal negative control: (A) antibody directed against the N-terminal (LS-C164143); (B) antibody directed against the C-terminal (ab84550); (C) antibody directed against phosphorylated merlin ser 518 (ab2478).

According to Spearman’s rank-order correlation, the expression of AB-N and AB-C was positively correlated (*P* =.017). Most of the 162 tumors from the TMA specimens had moderate to strong cytoplasmic immunoreactivity with all three merlin antibodies. None of the tumors were regarded as negative. The 20 WHO grade 2 meningiomas with known *NF2* status also had moderate to strong immunoreactivity with all three merlin antibodies. Merlin immunoreactivity and number of excluded cases are shown in Tables 4 and 5. Representative images for each antibody are demonstrated in Figure 1.

**Table 4.** Scoring of merlin immunoreactivity from the 162 patients with tumor specimens prepared as TMA.

Antibody	Negative	Weak	Moderate	Strong	Median [range]	Excluded
AB-N	0 (0%)	32 (19.8%)	93 (57.4 %)	34 (21.0 %)	2 [1-3]	3 (1.9%)
AB-C	0 (0%)	21 (13.0%)	42 (25.9 %)	97 (59.9 %)	3 [1-3]	2 (1.2%)
AB-P	0 (0%)	28 (17.3%)	83 (51.2%)	48 (29.6%)	2 [1-3]	3 (1.9%)

Abbreviations: AB-C, anti-NF2/merlin ab84550; AB-N, anti-NF2/merlin LS-C164143; AB-P, anti-NF2/merlin ab2478; TMA, tissue microarray.

**Table 5.** Scoring of merlin immunoreactivity for the 20 patients with known *NF2* status.

Antibody	Negative	Weak	Moderate	Strong	Median [range]	Excluded
AB-N	0 (0%)	0 (0%)	12 (60%)	6 (30%)	2 [2-3]	2 (10%)
AB-C	0 (0%)	1 (5%)	7 (35%)	12 (60%)	3 [1-3]	0 (0%)
AB-P	0 (0%)	2 (10%)	7 (35%)	9 (45%)	2.5 [1-3]	2 (10%)

Abbreviations: AB-C, anti-NF2/merlin ab84550; AB-N, anti-NF2/merlin LS-C164143; AB-P, anti-NF2/merlin ab2478.

### Relationships to clinicopathological features and *NF2* status

None of the applied antibodies had significant associations to WHO grade (*P *> 0.1), gender (*P *> 0.1), or tumor location (*P *> 0.1). The expression of merlin AB-C (*P* = .041) and merlin AB-P (*P* = .025) had significant relations to tumor subtype, yet none of the Bonferroni adjusted *P* values from Dunn’s test were significant for AB-C. However, the expression of merlin AB-P was significantly higher in meningothelial tumors compared to atypical meningiomas (adjusted *P = *.021). *NF2* status and 1p deletion were not significantly related to merlin immunoreactivity for any of the three applied antibodies (*P *> .2).

## DISCUSSION

We have investigated the immunohistochemical expression of merlin in 172 cases of meningiomas, including 20 cases with known *NF2* status, using antibodies directed against the AB-C, AB-N, and AB-P. All tumor cases were immunoreactive for the three antibodies. There were no significant associations of merlin immunoreactivity to WHO grade, gender, or tumor location. AB-C and AB-P had significant associations to tumor subtype following Kruskal-Wallis test, but only AB-P was found to be significantly higher in meningothelial tumors following the pairwise Dunn’s test and Bonferroni adjustment. Furthermore, merlin immunoreactivity had no association with *NF2* status or 1p deletion for any of the three applied antibodies.

### Merlin immunohistochemistry is a poor surrogate marker for *NF2* gene mutations

Previous studies have reported merlin immunoreactivity ranging from 25% to 100% of the cases.^9–12^^,^^14^^,^^15^^,^^19^ About 50% of sporadic meningiomas harbor *NF2* gene mutations, suggesting that these tumors have no or reduced merlin expression.^20^ However, our study reports merlin immunoreactivity in all cases of meningiomas. *NF2* mutation could result in expression of a nonfunctional merlin protein following nonsense, frameshift, or splice-site mutations,^20^ as also demonstrated by our findings. The discrepancy of observed and expected merlin immunoreactivity could be explained by expression of dysfunctional merlin protein following the mentioned *NF2* gene mutations. For instance, missense mutations still harbor a normal length of the merlin protein that could be detected by immunohistochemistry, although the protein has lost its normal function.^21–23^ Also, the choice of antibody could influence merlin immunoreactivity as antibodies targeting the N-terminal could be immunoreactive although the C-terminal is mutated.^22^ Antibodies targeting the N-terminal could also be prone to detect the N-terminal of ezrin, radixin, and moesin proteins, due to the structural similarity of these proteins to merlin, thereby decreasing the specificity of these antibodies to merlin.^5^^,^^21^ Phosphorylated merlin was detected in all our tumor cases, using AB-P. Phosphorylation of merlin, more specifically at serine 518, provides an active state of the protein, meaning that the merlin tumor suppressor activity is abolished.^5^^,^^24^  *NF2* status and 1p deletion had no impact on merlin immunoreactivity. The abundant expression of merlin also suggests no relation to the four molecular risk groups for WHO grade 1 and WHO grade 2 meningiomas, as described by the cIMPACT-NOW update 8.^3^ Accordingly, merlin immunoreactivity has no potential as a surrogate marker for *NF2* status in human meningiomas. Furthermore, as merlin is also expressed in some normal neural tissue, meningeal tissue and other brain tumors, the potential use of merlin as a diagnostic marker for meningiomas, like that of epithelial membrane antigen and somatostatin receptor 2, also appears limited.^5^^,^^10^^,^^24–26^

### Merlin immunoreactivity has sparse relations to clinicopathological features in human meningiomas

*NF2* gene mutations are known to be more frequent in some tumor locations, including the convexities of the brain, and as more frequent in fibrous and transitional subtypes.^1^^,^^18^^,^^27^ Also, compared to other genotypic variants, *NF2*-mutated meningiomas have larger tumor sizes and higher proliferation indexes.^20^ The latest cIMPACT-NOW update 8 further warrants the relation to higher malignancy grades and recommends that tumors with *NF2* oncogenic variants and/or monosomy of 22q with coincidental 1p deletion be classified as WHO grade 2 tumors.^3^ This could suggest an expected association of merlin expression to tumor subtype, WHO grade, and tumor location. Our study only found AB-P immunoreactivity to be significantly higher in meningothelial meningiomas, as could be explained by *NF2* mutations being less common in meningothelial meningiomas.^27^ None of the other applied antibodies were significantly associated with tumor location or tumor subtype. One could have expected less merlin immunoreactivity in convexity meningiomas, as *NF2*-mutated meningiomas are more frequent in this location.^1^^,^^28^ This was not the case, however, since merlin immunoreactivity was not linked to *NF2* status. According to Hanemann, there should be no correlation between merlin expression and WHO grade as mutations in merlin are assumed to be an initial gene defect and further tumor progression requires additional gene defects.^29^ In accordance with previous studies,^9^^,^^30^ we did not find any associations between merlin expression and gender.

### Strengths and limitations of the study

Strengths of this study are the large patient population with clinical data from one neurosurgical center. The use of three polyclonal antibodies targeting different epitopes of merlin ensured high detection sensitivity. Potential limitations are the retrospective nature of the study and the inherent challenges of immunohistochemistry. The use of polyclonal antibodies could imply lower antibody specificity compared to that of monoclonal antibodies.^31^ Reclassification of the included tumors to the WHO 2021 classification could increase the number of WHO grade 3 tumors. Still, loss of *CDKN2A/B* and *TERT* promotor mutations are rare mutations and not believed to have significant impact to our findings.^4^  *NF2* gene analyses on all included tumors would have further clarified the relation between genetic aberrations and merlin expression. Furthermore, the present study does not include investigations of any downstream proteins that could be used as an alternative to merlin for identification of *NF2*-mutations.

In conclusion, our study found an abundant expression of merlin for all applied antibodies. Due to the frequency of *NF2* gene mutations in meningiomas, we expected reduced or lost merlin expression in *NF2*-mutated meningiomas. AB-P was significantly higher in meningothelial meningiomas; otherwise, merlin expression was not related to WHO grade, gender, tumor location, or tumor subtype. The diagnostic role of merlin immunoreactivity appears limited. Accordingly, we conclude that merlin immunoreactivity is not sufficient as a surrogate marker for *NF2* mutations. However, as previous studies report variation in merlin immunoreactivity, further studies are needed to clarify the impact of merlin immunoreactivity.
